# An Italian Adaptation of the Brief Resilient Coping Scale (BRCS) and Attitudes During the Covid-19 Outbreak

**DOI:** 10.3389/fpsyg.2021.641213

**Published:** 2021-07-01

**Authors:** Mike Murphy, Andrea Lami, Carmen Moret-Tatay

**Affiliations:** ^1^School of Applied Psychology, University College Cork, Cork, Ireland; ^2^European Asylum Support Office, Rome, Italy; ^3^MEB Laboratory, Universidad Católica de Valencia San Vicente Mártir, Valencia, Spain; ^4^NESMOS, Sapienza University of Rome, Rome, Italy

**Keywords:** Brief Resilient Coping Scale, multigroup analysis, attitudes, Covid-19, psychometric

## Abstract

Resilience has attracted the interest of the scientific community during the Covid-19 outbreak, as a protective factor in mental health. As the migrant population arguably has one of the most vulnerable profiles in the current health crisis, the aim of this study is to assess the psychometric properties of the Italian version of the Brief Resilient Coping Scale (BRCS) across native and migrant residents in Italy, and to compare scores across these two populations. Other personal attitudes to the current restrictions were considered. Preliminary psychometrics were tested in a version of the translated instrument with an independent sample. A second independent sample was used to analyse the differences between migrant and native adults. The results showed no differences between the new version and the previous Spanish adaptation or the original instrument. Moreover, no differences were found between the migrant and non-migrant group. BRCS scores were predicted by attitudes toward Covid-19 but not by migrant or native group. These results suggest that the BRCS may be a useful tool to measure resilience in Italy at time of pandemic, irrespective of cultural differences.

## Introduction

Resilience is defined as the capacity to overcome adversity but also to be transformed by these experiences (Herrman et al., 2011). Two ways of understanding resilience have emerged: as a personality trait (Fino et al., 2020); or as a consequence of coping with experiences, mixed with a trait component (Parsons et al., 2016; Ye et al., 2020). Studies during the current pandemic have shown the direct impact of stress, arising from the circumstances, on mental health (Bozdag and Ergün, 2020; Ye et al., 2020). Contemporary society is currently facing an urgent psychological need for support in an unprecedented health crisis worldwide, and resilience might be a protective key in this situation (West et al., 2020). In this scenario, an interesting underlying topic is related to how this concept varies not only across cultures, but also contexts.

Previous disaster literature, related to such occurrences as Hurricane Katrina, the Haitian earthquake of 2010, and the Fukushima nuclear event of 2011, has shown an inverse relationship between resilience and distress (Osofsky and Osofsky, 2013; Blanc et al., 2016; Kaye-Kauderer et al., 2019). Studies during the current pandemic have shown the direct impact on the mental health of individuals of stress arising from the circumstances (Burrai et al., 2020; Roma et al., 2020; Pérez-Mengual et al., 2021). It is clear that society is currently facing the urgent psychological need for support in an unprecedented health crisis worldwide, and resilience might be a key protective factor in this situation (West et al., 2020). In this scenario, an interesting underlying topic is related to how this concept varies not only across cultures, but also contexts.

Many scales have been developed for the purpose of measuring resilience, demonstrating good psychometric properties, both in reliability and validity. Some of the most widely-used scales are the Resilience Scale (Wagnild and Young, 1993), the Dispositional Resilience Scale (Bartone, 2007) and the Brief Resilient Coping Scale (BRCS; Sinclair and Wallston, 2004). The BRCS has proven robust, with adequate levels of reliability and validity. It consists of 4 items, with responses on a 5-point Likert scale, and with item scores added to yield a single total score. Its main benefit is that it offers a one-dimensional structure composed of four items, which is easy to apply and has demonstrated good psychometric properties. Although the BRCS has been translated into different languages and validated for use with specific populations (Tomás et al., 2012; Moret-Tatay et al., 2015; López-Pina et al., 2016; Rice and Liu, 2016; Heinen et al., 2017) demonstrating that it is a valuable assessment instrument, its psychometric properties have not been thoroughly examined among Italian-speaking populations. This country was one of the first in Europe to suffer the effects of Covid-19, and also to adopt restriction measures and lockdown. It is possible that examination of differences in resilience between native Italians and potentially more vulnerable and less integrated migrants at a time of pandemic might provide some support for one or other of the trait and state-trait explanations of resilience.

As expected by its complexity, there are many variables that might underlie the resilience concept. According to the literature, resilience may be a key variable in understanding stress experienced due to the Covid-19 effects across ages (Pearman et al., 2020; Vahia et al., 2020). Moreover, gender differences seem to appear across profiles and cultures (Moret-Tatay et al., 2016; Iimura and Taku, 2018; Portnoy et al., 2018), and even a moderator effect of gender has been described (Zhang et al., 2018). Migrant status may also play a role in predicting resilience if the state-trait explanation is correct.

As has occurred in other crises or catastrophes, migrants may be particularly vulnerable to both direct and indirect effects of Covid-19. Meanings and functions of social networks which are crucial for them (Sundvall et al., 2021) may be particularly affected. Moreover, it should be noted that problems related to adequate health care, economic well-being, religious expression, living and working conditions, and inclusion in host communities, among others, often vary according to migration status (Cetrez, 2011; Ledoux et al., 2018; Aragona et al., 2020). Conversely, experience of successful coping with adversity experienced in the migration process might be expected to increase resilience. In this case, Italy is considered a country with a prolonged immigration transit within frontier countries in Europe (Assirelli et al., 2019; Giordano et al., 2019). Analyzing differences between the Italian and migrant samples might be considered an opportunity to address the State-Trait dimension in resilience.

The aim of this research is to study the psychometric properties of the Italian BRCS adaptation over different groups related to personal characteristics, such as age, gender and country of origin. This might shed light on the construct's nature across individuals from different cultures and levels of vulnerability. Moreover, attitudes toward the Covid-19 outbreak are also considered, as positive attitudes have been found to be predictors of adherence to health regulations and guidance (e.g.„ vaccination) in previous literature (Cordina and Lauri, 2021).

## Methods

### Sample

Two independent samples were selected. The first sample was a group of university students in an international mobility programme. A total of 20 Italian students (with an average age of 23.15 years, *SD* = 5.54), with high proficiency in Spanish and English, as well as 22 Spanish students (with an average age of 24.50 years, *SD* = 4.36) with high proficiency in Italian and English participated in this first study. None of these students has a level lower than B2 in the languages under study (according to CEFR Language Levels). For the Italian subsample, 60% were women, and for the Spanish one 81.8%.

A second independent sample of 526 participants volunteered to take part in this study. They were divided into two groups: migrants and non-migrants. The first group was composed of 105 participants, of whom 29.5 % were male and 70.5 % were female. A majority (69.5%) had migrated from European countries, while 30.5% came from a non-European country, as described in Appendix A. The average age was 30.2 years (*SD* = 8.42), with an age range of 19-62. The non-migrant group was composed of 421 participants, of whom 36.8% were male and 63.2% were female. The average age was 32.35 years (*SD* = 13.81), with an age range of 18–81.

### Procedure

Data was collected online through Qualtrics software, which was distributed by different communication applications. This study was approved by the ethics committee of the School of Applied Psychology of UCC on April 6, 2020. The study was carried out in April 2020. Non-probability sampling was employed, under an incidental sample. The inclusion criteria for the first and second subsample was very similar except for the language requirements described in participant section. Thus, the criteria for the second subsample were: (i) the participants had to be 18 years old or over, (ii) Speak fluent Italian and be resident in Italy for the second study (and Italy or Spain in the first one), and (iii) consent to participate, in accordance with the Declaration of Helsinki. Participation was completely voluntary and could be withdrawn at any time.

Additionally, for the first sample, and as described in previous literature procedures (Sousa and Rojjanasrirat, 2011), participants were asked to answer the Italian version BRCS, and were asked to supply socio-demographic data as a distractor. Secondly, they had to answer to the BRCS Spanish scale used for the translation, and afterwards a new distractor block of questions about their language proficiency was presently. Lastly, participants had to answer the original BRCS scale in English.

### Instruments

Sociodemographic data, as well as questions developed to measure attitudes toward the Covid-19 outbreak on a Likert scale from 1 to 10 (as reported in Murphy and Moret-Tatay, 2021), were employed in an isolated way. These questions are sorted as follows:

- I fear people with contagious diseases
  *Temo le persone con malattie contagiose*
- I think my sense of belonging to Italy has increased since the outbreak began *Penso che il mio senso di appartenenza all'Italia sia aumentato dall'inizio del Covid-19*.
- I consider that I have correctly informed myself about Covid-19
  *Ritengo di essermi informato/a correttamente sul Covid-19*.

Lastly, an Italian adaptation of the BRCS was developed. In order to facilitate conducting this research with an Italian-speaking sample, a back translation of the previous Spanish adaptation (Moret-Tatay et al., 2015) was carried out, in accordance with procedures described in previous literature (e.g., Chen et al., 2009; Muñiz et al., 2013). To properly consider linguistic and cultural factors during the adaptation, several independent tranlations were reviewed by a committee of four with knowledge of Italian language and culture. One bilingual researcher translated, another back-translated, and all items were reviewed by authors to confirm meaning and comparability. Considering the strong similarities between Italian and Spanish cultures, it is considered unlikely that important differences in the meaning and understanding of resilience will appear. Nevertheless, an independent sample of Italian university students and Spanish one answered to the three BRCS versions and their results were compared.

The resulting scale is presented below:

- I look for creative ways to alter difficult situations
  *Tendo a cercare modi creativi per affrontare situazioni difficili*.
- Regardless of what happens to me, I believe I can control my reaction to it
  *Non importa quel che mi succede, sono fiducioso/a di poter controllare la mia reazione*
- I believe I can grow in positive ways by dealing with difficult situations
  *Penso di poter imparare cose positive quando devo affrontare situazioni difficili*.
- I actively look for ways to replace the losses I encounter in life
  *Ricerco attivamente modi per sostituire le perdite che mi capitano nella vita*.

### Data Analysis

All statistical analyses were performed using the software IBM SPSS 21 and AMOS 21. Assumptions were checked to ensure the appropriateness of factor analysis—high sample size, multivariate normality, linearity and correlation between variables (Tabachnick and Fidell, 1989). We checked for internal consistency of the scale through mean inter-item correlation, Cronbach's Alpha, and assessed items of homogeneity; KMO index and the Bartlett test of sphericity (Kaiser, 1974).

After a descriptive approach and comparisons of means through a t-test for independent samples, a confirmatory factor analysis (CFA), accompanied by goodness of fit indices, was conducted. Confirmation of the adequacy of the model used absolute fit indices; the chi-square statistic X^2^ (Saris and Stronkhorst, 1984); the comparative fit index (CFI) with a reference value of 0.90 (Bollen, 1989), and, within parsimony adjustment indices, the error of the root mean square approximation (RMSEA), for which the smaller the value the better the fit, the reference value being 0.06 (Hu and Bentler, 1999). Secondly, a multigroup structural model was done to fully understand the multivariate relationships and compare both samples. This analysis offers, from the field of structural equation modeling techniques, a hierarchical procedure to estimate parameters for determination of the model as well as the associated standard error, comprising variances and the coefficients related to relationships between variables or measures (Bentler, 1992). This hierarchy starts by testing the factorial homogeneity structure across groups, from a stage where all parameters do not need to be equal to a stage where they do. Pearson's correlation and linear regression in predicting fear of Covid-19 were carried out.

## Results

First, a non-parametric approach was carried out across the scores on BRCS for the first sample of 20 and 22 international students. As depicted in Appendix B, scores and correlations were very similar across the Italian version of the BRCS, and both the Spanish and English versions. The Friedman test showed no statistically significant differences, as expected.

With regards to the second independent sample composed by migrant and non-migrant resident in Italy depicted an α = 0.69. Table 1 depicts the descriptive data for the main variables of interest. Levene's test on equality of variances was satisfactory (*p* > 0.05) across groups. While score variables were slightly higher for the migrant group, differences did not reach the statistical significance in a *t*-test for independent samples.

**Table 1:** Descriptive analysis on the main variables of interest (*SD* = standard deviation), and *t*-test for independent samples across groups.

	**Group**	**N**	**Mean**	**SD**	***P***	**95% CI (Lower-Upper)**
People living with (living)	Non-migrant	419	2.46	1.48	0.05	(−0.43; −0.002)
	Migrant	104	2.81	2.15		
Fear of contagious diseases (fear)	Non-migrant	420	6.14	2.51	0.27	(−0.009; 0.33)
	Migrant	105	5.83	2.51		
Sense of belonging (belonging)	Non-migrant	421	5.10	2.79	0.77	(−0.24; 0.18)
	Migrant	105	5.19	2.96		
Correctly informed (informed)	Non-migrant	421	7.84	1.61	0.19	(−0.35; 0.007)
	Migrant	105	8.07	1.63		
Resilience (BRCS)	Non-migrant	421	14.23	2.89	0.24	(−0.34; 0.08)
	Migrant	105	14.60	2.74		

Cronbach's alpha of the BRCS scale showed an adequate values for both migrant and non-migrant samples (0.062 and 0.71, respectively), as well as mean inter-item correlation for the migrant (from 0.29 to 0.51) and non-migrant (from 0.49 to 0.51) samples. Although the development of a confirmatory factor analysis (CFA) may be a matter of debate as a unidimensional factor is expected from 4 items, it was conducted so as to be consistent with previous literature (Tomás et al., 2012; Moret-Tatay et al., 2015). The CFA confirmed the existence of a single factor for the whole dataset: χ^2^(2) = 6.26; χ^2^/*df* = 3.13; *p* = 0.04; CFI = 0.98; RMSEA = 0.06. A multigroup analysis was carried out to test invariance across, both groups. Table 2 depicts acceptable values up to and including structural covariance level.

**Table 2:** Analysis of invariance across migrant and non-migrant groups.

**Model**	**χ^2^**	**df**	**χ^2^/df**	**CFI**	**RMSEA**	**Δχ^2^**	**Δdf**	**Decision**
Unconstrained	7.56	4	1.89	0.989	0.041	-	-	-
Measurement weights	12.07	7	1.72	0.984	0.037	4.51	3	Accept
Measurement intercepts	12.61	8	1.58	0.986	0.033	0.54	1	Accept
Structural covariances	15.70	12	1.31	0.989	0.024	3.09	4	Accept

A Pearson's coefficient was carried out on the variables of interest for the whole dataset. As depicted in Table 3 several relationships were found, being the Sense of belonging and Fear to contagious diseases the strongest one.

**Table 3:** Pearson Correlations on the variables of interest for the whole dataset.

	**Age**	**Fear**	**Belonging**	**Informed**	**BRCS**
Age	—				
Fear	0.204^***^	—			
Belonging	0.163^***^	0.338^***^	—		
Informed	−0.038	0.142^**^	0.085	—	
BRCS	0.142^**^	−0.080	0.095^*^	0.044	—

* *p < 0.05,*

** *p < 0.01,*

*** *p < 0.001*.

When the Pearson's coefficient was carried out exclusively on the migrant group, BRCS correlated with Informed (*r* = 0.24; *p* < 0.01), Informed with Fear (*r* = 0.22; *p* < 0.01), Belonging with Fear (*r* = 0.43; *p* < 0.001), and Fear with Age (*r* = 0.23; *p* < 0.01). When the Pearson's coefficient was carried out exclusively on the non-migrant group, BRCS correlated with Age (*r* = 0.15; *p* < 0.001), Informed with Fear (*r* = 0.12; *p* < 0.01), Belonging with Fear (*r* = 0.31; *p* < 0.001), Fear with Age (*r* = 0.20; *p* < 0.001), and Belonging with Age (*r* = 0.18; *p* < 0.001).

Lastly, a regression analysis was carried out on the prediction of fear of contagious diseases. The model reached statistical significance: *F*_(6, 518)_ = 19.86; MSE = 103.56; *R*^2^ = 0.19; *p* < 0.001. As depicted in Table 4, migration status did not reach the statistical significance, while the rest of variables did.

**Table 4:** Linear regression analysis on the prediction of Fear in the whole dataset, which reached statistical significance: *F*_(6, 518)_ = 19.86; MSE = 103.56; *R*^2^ = 0.19; *p* < 0.001.

**Model**		**B**	**SE**	**β**	***t***	***P***
1	(Intercept)	2.735	0.874		3.129	0.002
	Age	0.037	0.008	0.190	4.642	<0.001
	Gender	0.642	0.213	0.122	3.009	0.003
	BRCS	−0.108	0.036	−0.122	−3.014	0.003
	Belonging	0.265	0.036	0.298	7.310	<0.001
	Informed	0.209	0.062	0.135	3.377	<0.001
	Group	−0.305	0.251	−0.049	−1.216	0.224

## Conclusions and Discussion

The aim this research was to examine the psychometric properties of the Italian adaptation of the BRCS, for migrant and non-migrant participants. Moreover, variables such as age, sex, attitudes toward the Covid-19 outbreak and country of origin were also considered in the prediction of fear of contagious diseases. The main results are described as follows: (i) the Italian BRCS adaptation depicted good psychometric properties for both migrant and non-migrant groups; (ii) The multigroup analysis pointed to a high invariance across groups; (iii) A relationship of BRCS was found with Informed in the migrant group and Age in the non-migrant; (iv) Migrant group did not predict Fear.

The current results suggest that the BRCS Italian adaptation may be a useful tool to measure resilience in both the Italian population, and the resident migrant population. Not only did psychometric properties show adequate indices, but invariance also reached a high level of parameters across samples. In an applied level, one should bear in mind that the short number of items makes it an easier solution in terms of time of application, which might also avoid fatigue from respondents when several variables want to be measured.

Even if migrants can be considered a vulnerable group, no differences were found with the non-migrant group in terms of resilience. This could be related to the specific characteristic of the current sample. Purposes of the migration were not canvassed, but according to sociodemographic data a student profile predominates. This issue needs clarification in further research, as living conditions, as well as economic status, might be an important predictor of the variables under study. Nevertheless, the results seems to partially support the hypothesis that resilience might contain stable components described as traits in the literature (Fino et al., 2020). Future lines of research should address the role of living conditions in this scenario. Moreover, longitudinal research might shed light on the mixed State-Trait nature.

Gender and age predicted fear of contagious diseases as expected from previous literature (Özdin and Bayrak Özdin, 2020). Literature has suggested that women's traditional role as main caregivers, combined with working from home, may be partly responsible (Gausman and Langer, 2020). This blurring situation might lead to increased anxiety, depicting a context-specific vulnerability, which is of interest for future lines of research. Moreover, older adults might experience higher fear, and not anxiety or loneliness, because of isolation and health conditions (Luchetti et al., 2020); this should also be addressed in future lines of research.

Effects of attitudes toward Covid-19 were also measured as previously approached in the literature. This is of interest to understand the role of resilience in the current pandemic. Sense of belonging to Italy was correlated to fear of contagious diseases for both groups. This result might support traditional theories which explains how, in times where death awareness emerge, individuals tend to strengthen their self-esteem to reduce anxiety against death (Pyszczynski et al., 1999). Moreover, this can also lead to a search for cohesion between people who share similar world views and hostility toward those with alternative world views (Su and Shen, 2021). Regarding feeling well-informed about Covid-19, it is described in the literature that ambiguous information is related to fear (Lissek et al., 2006). In this case, the migrant group showed a correlation between this variable and Resilience. This might reflect a special sensibility in the migrant group to look for information that calms them.

The main limitations that arise is that the sample was selected through non-probability sampling, which can introduce distortions in the results. Moreover, data was recruited in a self-report way. Thus, biases might occur with regards to a high component of self-perception in the data. With regards to personal characteristics, there is a significantly higher number of women than men. Lastly, some variables related to attitudes were measured though a single item in a Likert format scale of 10 points. These were selected as procedures from previous literature (Boot et al., 2015; Moret-Tatay et al., 2019) considering these items have face validity and capture the main ways in which people could experience the restrictions effects, such as prevailed when the data were gathered.

## Data Availability Statement

The raw data supporting the conclusions of this article will be made available by the authors.

## Ethics Statement

The studies involving human participants were reviewed and approved by UCC approval on April 2020. The patients/participants provided their written informed consent to participate in this study.

## Author Contributions

MM and CM-T drafted the manuscript. AL and CM-T focused on the back translation process. All authors have substantial contributed to the conception and design of the work, the acquisition, analysis, and interpretation of data for the work and approved the final version of the manuscript.

## Conflict of Interest

The authors declare that the research was conducted in the absence of any commercial or financial relationships that could be construed as a potential conflict of interest.

## Appendix

**Appendix A:** Descriptive analyses on migrant group (non-eu with a sample size of *N* = 32 or 30.5%, and eu with an *N* = 73 OR 69.5%).

**List of countries**		**BRCS**	**Living with**	**Fear**	**Sense of belonging**	**Well-informed**
Albania (*n* = 3)	Mean	15.33	4.33	9.00	7.67	9.00
	*SD*	0.58	1.15	1.00	2.52	1.73
Argentina (*n* = 1)	Score	16.00	5.00	7.00	5.00	10.00
Brazil (*n* = 3)	Mean	15.00	3.00	7.67	5.67	8.67
	*SD*	2.00	1.73	3.21	4.04	1.15
Cabo Verde (*n* = 1)	Score	15.00	2.00	5.00	5.00	4.00
China (*n* = 4)	Mean	16.25	2.75	5.75	3.00	7.25
	*SD*	2.75	1.50	2.22	2.31	2.36
Colombia (*n* = 3)	Mean	12.33	3.00	8.00	7.67	7.00
	*SD*	1.15	1.00	2.00	1.53	2.00
Croatia (*n* = 1)	Score	15.00	2.00	4.00	1.00	4.00
Cuba (*n* = 1)	Score	13.00	1.00	3.00	1.00	10.00
Equator (*n* = 1)	Score	19.00	3.00	6.00	9.00	9.00
France (*n* = 2)	Mean	14.50	3.00	7.00	5.50	9.00
	*SD*	2.12	1.41	2.83	6.36	0.00
Greece (*n* = 1)	Score	14.00	4.00	6.00	7.00	8.00
Republic of Ireland (*n* = 2)	Mean	12.50	2.50	9.50	10.00	7.00
	*SD*	0.71	2.12	.71	.00	1.41
Latvia (*n* = 1)	Score	12.00	4.00	7.00	6.00	8.00
Mexico (*n* = 1)	Score	13.00	2.00	8.00	8.00	9.00
Morocco (*n* = 6)	Mean	16.67	3.17	5.00	5.33	8.83
	*SD*	1.21	1.17	2.00	3.33	1.33
Peru (*n* = 1)	Score	16.00	4.00	4.00	5.00	7.00
Poland (*n* = 6)	Mean	14.50	2.83	6.33	5.33	8.67
	*SD*	3.39	0.98	2.94	3.67	1.51
Portugal (*n* = 3)	Mean	16.33	2.00	6.67	5.67	9.33
	SD	2.52	1.00	2.31	4.04	1.15
Romania (*n* = 10)	Mean	12.90	2.60	4.90	6.00	8.20
	*SD*	4.28	0.70	3.00	2.79	1.62
Russian Federation (*n* = 2)	Mean	14.50	4.00	7.00	3.50	7.00
	SD	0.71	1.41	2.83	0.71	2.83
Spain (*n* = 45)	Mean	15.02	2.75	5.42	4.71	8.07
	*SD*	2.33	3.05	2.55	2.82	1.57
Turkey (*n* = 2)	Mean	11.50	3.00	6.00	4.00	6.50
	*SD*	4.95	1.41	2.83	4.24	0.71
Ukraine (*n* = 3)	Mean	11.67	1.67	4.67	4.67	7.33
	*SD*	3.79	0.58	0.58	2.52	0.58
United Kingdom of Great Britain and Northern Ireland (*n* = 2)	Mean	12.50	2.50	6.00	5.00	8.50
	*SD*	3.54	0.71	2.83	2.83	0.71

*Migrant education level, 1% had not completed primary education, 1% had completed primary education; 12.4% had completed secondary level studies and 87.5% had continued to higher education. A total of 16.2% reported still attending their place of work, 10.5% were working from home, 5.7% could not carry out their work at that time, 1% were homemakers, 61% full-time students, and 5.7% unemployed since before the pandemic began. Non-migrant education level: 1% had completed primary education; 31.6% had completed secondary level studies and 67.5% had continued to higher education. A total of 17.6% reported still attending their place of work, 11.6% were working from home, 6.7% could not carry out their work at that time, 3.1% were retired, 0.5% were homemakers, 53.4% full-time students, and 7.1% unemployed since before the pandemic began*.

**Appendix B:** Socres on the three bRCS language versions employed for the first sample (sd in brackets).

**Nationality**	**BRCS**	**Item 1**	**Item 2**	**Item 3**	**Item 4**
Italian (*n* = 20)	Spanish	4.10 (0.96)	3.5 (1.14)	3.95 (0.82)	3.80 (1.15)
	English	4.15 (0.93)	3.5 (1.14)	4	3.85 (1.13)
	Italian	4.10 (0.97)	3.40 (1.09)	4 (0.86)	3.8 (1.15)
Spanish (*n* = 22)	Spanish	4.04 (0.78)	3.68 (0.94)	3.90 (0.92)	4 (0.92)
	English	4.09 (0.81)	3.68 (0.94)	3.90 (0.92)	3.95 (0.89)
	Italian	4.09 (0.81)	3.72 (0.98)	3.98 (0.95)	4 (0.92)

*Correlation across items for the Italian version in Italian participants ranged from 0.604 to 0.831, and for the Spanish participants from 0.617 to 0.826*.

*Correlation across items for the Spanish version in Italian participants ranged from 0.491 to 0.769, and for the Spanish participants from 0.679 to 0.784*.

*Correlation across items for the English version, and original one, in Italian participants ranged from 0.526 to 0.815, and for the Spanish participants from 0.682 to 0.784*.
